# Moisture dipole over the Tibetan Plateau during the past five and a half centuries

**DOI:** 10.1038/ncomms9062

**Published:** 2015-08-21

**Authors:** Qi-Bin Zhang, Michael N. Evans, Lixin Lyu

**Affiliations:** 1 State Key Laboratory of Vegetation and Environmental Change, Institute of Botany, Chinese Academy of Sciences, 20 Nanxincun, Haidian District, Beijing 100093, China; 2 Department of Geology & ESSIC, University of Maryland, Geology Bldg (#237), Rm 1120, College Park, Maryland 20742, USA

## Abstract

The South Asian Monsoon and mid-latitude Westerlies are two important controls on Tibetan Plateau (TP) fresh water resources. Understanding their interaction requires long-term information on spatial patterns in moisture variability on the TP. Here we develop a network of 23 moisture-sensitive tree-ring chronologies from major juniper forests in a north–south transect on the eastern TP. Over the past five and a half centuries, we find that these chronologies cluster into two groups, North and South, of ∼33° N. Southern and northern regional chronology subsets are positively and significantly correlated with May–June Palmer Drought Severity Indices (PDSI). The meridional moisture stress gradient reconstructed from these data suggests substantial stochastic variation, yet persistent moisture stress differences are observed between 1463–1502 CE and 1693–1734 CE. Identification of these patterns provides clues linking them with forced or intrinsic tropical–extratropical interactions and thus facilitates studies of interannual–decadal dipole variations in hydroclimate over the TP.

## Abstract (web summary)

Controls on the Tibetan Plateau fresh water resources are poorly understood. Here, the authors develop juniper tree-ring chronologies representing over 500 years and show that northern and southern subsets exist due to variations in hydroclimate over this time period.

## Graphical abstract

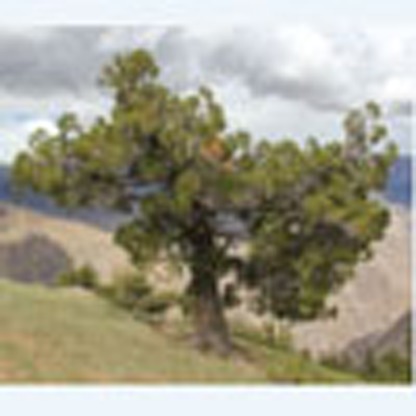

Known as the ‘Water Tower' in central Asia^1^, the Tibetan Plateau plays an important role in regulating water resources in and beyond the plateau, thus affecting the well-being of billions of people. Variations in moisture supply to the Tibetan Plateau are mainly influenced by tropical–extratropical interaction, but our current understanding of this interaction is inconsistent^2^,^3^,^4^. Studies of the oxygen and deuterium isotopic composition of modern precipitation and river water indicate that the source of summer precipitation in the southern plateau is different from that in the northern plateau, with the South Asian Monsoon dominating the south and mid-latitude Westerlies dominating the north^5^,^6^,^7^. On the other hand, studies of meteorological data and glacier mass balance reveal an out-of-phase pattern in moisture conditions on the southern and northern Tibetan Plateau and an influence of moisture change on the southern plateau by mid-latitude dynamics^8^,^9^,^10^. Examination of paleoclimate records of ice cores, speleothems and lake sediments with quinquennial to millennial-scale observational resolution and chronological control have shown a broad pattern of inferred moisture variations and related interplay between tropical and extratropical moisture sources^11^,^12^,^13^. Further understanding of these tropical–extratropical interactions requires independent, annually resolved estimates of warm season Tibetan Plateau moisture variability on both the southern and the northern Tibetan Plateau to fill the gaps between the short but highly resolved instrumental records and the coarser resolution paleoclimatic observations, so that the projected anthropogenic forced variation^14^ can be placed into the context of the unforced and intrinsic variations on decadal to centennial timescales^15^.

Here we investigate the spatial pattern and temporal evolution of moisture conditions on the Tibetan Plateau using a network of annually resolved and calendar-year dated tree-ring width chronologies. Our observations are new and independent of those used to develop the Monsoon Asia Drought Atlas (MADA)^16^, which provides gridded reconstruction of June–August Palmer Drought Severity Index (PDSI)^17^—an index of cumulative effect of atmospheric moisture supply and demand at the surface over the past 700 years. Our tree-ring chronologies are from data-sparse forested regions of the eastern Tibetan Plateau, in a transect spanning 29–37° N, and are developed from moisture-sensitive junipers (*Juniperus spp.*). Relative to the MADA, these new records significantly increase the spatial coverage of data on the Tibetan Plateau, and facilitate precise identification of climatically important events.

Our study demonstrates that the early growing season moisture conditions are not meridionally uniform on the Tibetan Plateau for the past five and a half centuries. In particular, we identify two states of prolonged south–north moisture dipole in 1,463–1,502 CE and 1,693–1,734 CE, which might reflect a change in Tibetan Plateau hydroclimate related to tropical–extratropical interactions during the Little Ice Age^18^,^19^. Our records may provide further basis for mechanistic understanding of the response of the Tibetan Plateau moisture delivery to changes in external radiative climate forcing within these two periods, and for validating the statistics of the intrinsic variations in Tibetan Plateau moisture conditions as represented in climate models^14^,^20^.

## Results

### Development of the moisture-sensitive tree-ring network

The elevations of the forests for sample collection were between 3,200 and 4,700 m.a.s.l. At each site, trees that were presumably of old age and sensitive to climate change were selected for sampling. The practical selection of these trees was based on tree morphology and local conditions, such as presence of stripbark, large-diameter stems, growth in coarse-textured soil on a slope and minimal competition from neighbouring trees. High-quality increment cores were collected from at least 20 individual trees at each site. Tree-ring widths of these samples were measured, crossdated and composited into a site chronology following standard dendrochronological techniques (see Methods section).

**Figure 1:**
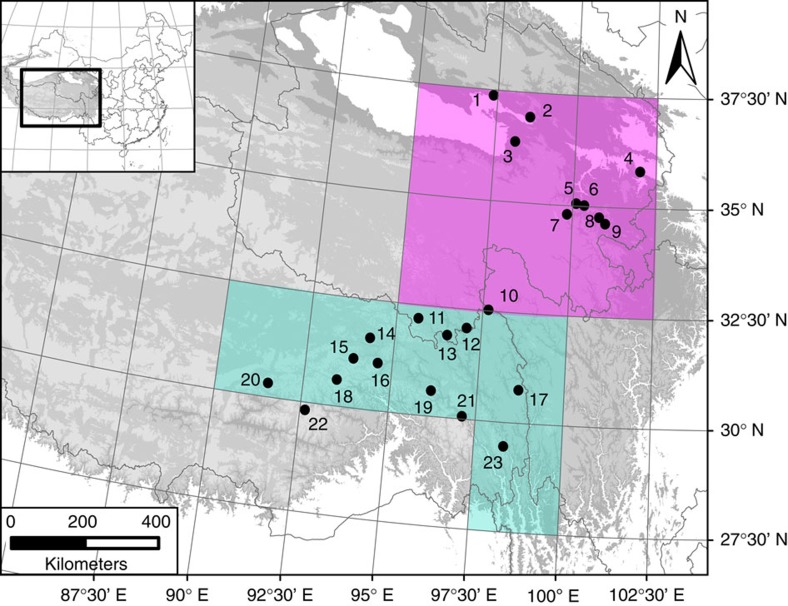
Location of the 23 sites for tree-ring network studies on the Tibetan plateau. The six grids in red represent the northern region PDSI data grids and the five grids in green represent the southern region PDSI data grids used to develop the regional reconstruction targets. The site numbers are in the order from north to south and the site information is listed in Supplementary Table 1.

To date, we have developed tree-ring width chronologies at 32 sites in the north–south transect. Statistical analysis of the growth–climate relationship at each study site indicates that chronologies at 23 sites (Fig. 1) are sensitive to gridded local May–June PDSI^17^ (Supplementary Table 1). Results from realistically nonlinear and multivariate forward modelling of tree-ring width variations^21^ with specified May–June growing season are also consistent with observed tree-ring widths, supporting the statistical-empirical interpretation of growth variations as indicative of May–June moisture variations (Supplementary Fig. 1). These 23 site tree-ring chronologies were therefore selected to form a tree-ring network for further analysis of large-scale moisture variability on the Tibetan Plateau. Among these 23 site chronologies, 16 are newly reported here and 14 are more than five centuries long.

### Tree-ring spatial structure and regional chronologies

**Figure 2:**
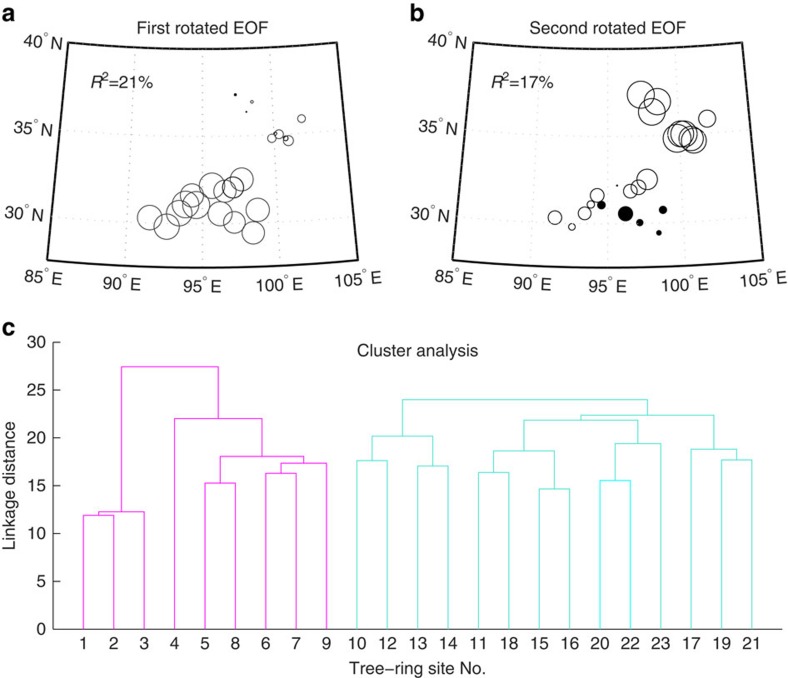
Spatial structure of the 23 site chronologies in the common period 1,753–2,000 CE. The size of circles represents value of loadings for sites in the first rotated EOF (**a**) and the second rotated EOF (**b**; the filled circle represents value of opposite sign). Cluster analysis shows linkage relations of the northern sites (coloured in pink) and southern sites (coloured in green; **c**). The tree-ring site numbers are arranged in the order of latitude from north to south and are consistent with those in Fig. 1 and Supplementary Table 1.

Rotated empirical orthogonal function (EOF) analysis^22^ was conducted for the 23 site chronologies in the common period 1753–2000 to identify their spatial structure and leading time series expansions. These chronologies were standardized to minimize sensitivity of the results to variance differences between series. The loadings of the first two EOFs show that the northern 9 site chronologies behave differently from the southern 14 sites with a dividing line at ∼33° N (Fig. 2a,b). The same two groupings of the site chronologies are also revealed independently by cluster analysis (Fig. 2c). Therefore, the 9 site chronologies in the northern group and the 14 site chronologies in the southern group were standardized and averaged in each group to derive two regional chronologies representing the northern and southern regions of the eastern Tibetan Plateau, respectively. These regionally averaged chronologies are in high agreement with the time series of the first and second principal components of the EOF analysis (Supplementary Fig. 2).

Because the length of each site chronology was different, we truncated the regional chronologies in years when the number of component site chronologies was below five. Thus, the north (south) regional chronology spans 1,442–2,005 CE (1,451–2,006 CE). The correlation between the average of the five longest chronologies and the regionally averaged full chronologies in the period 1753–2000 is *r*=0.95 (*N*_eff_=243, *P*<0.001) for the northern region and *r*=0.89 (*N*_eff_=227, *P*<0.001) for the southern region, indicating that the longest chronologies, although containing fewer tree-ring width observations, nevertheless contain the same signal as in the better replicated but shorter chronologies. The spatial structure of these 10 longest chronologies show that the division at ∼33° N is generally a feature of the past five and a half centuries (Supplementary Fig. 3).

### Reconstruction of May–June PDSI variability

**Figure 3:**
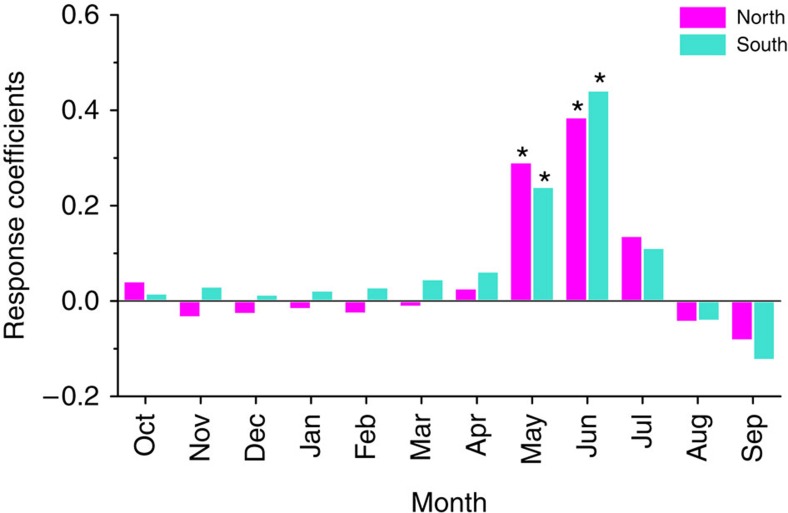
Relationships between regional tree-ring chronologies and monthly PDSI. Response coefficients were calculated for months from October of prior growth year to September of growth year in 1953–2005 for the north (represented by pink bars) and south (represented by green bars) regions of eastern Tibetan Plateau. Response coefficients significant at *P*<0.05 (as tested by 1,000 bootstrap replications) are denoted by stars.

**Figure 4:**
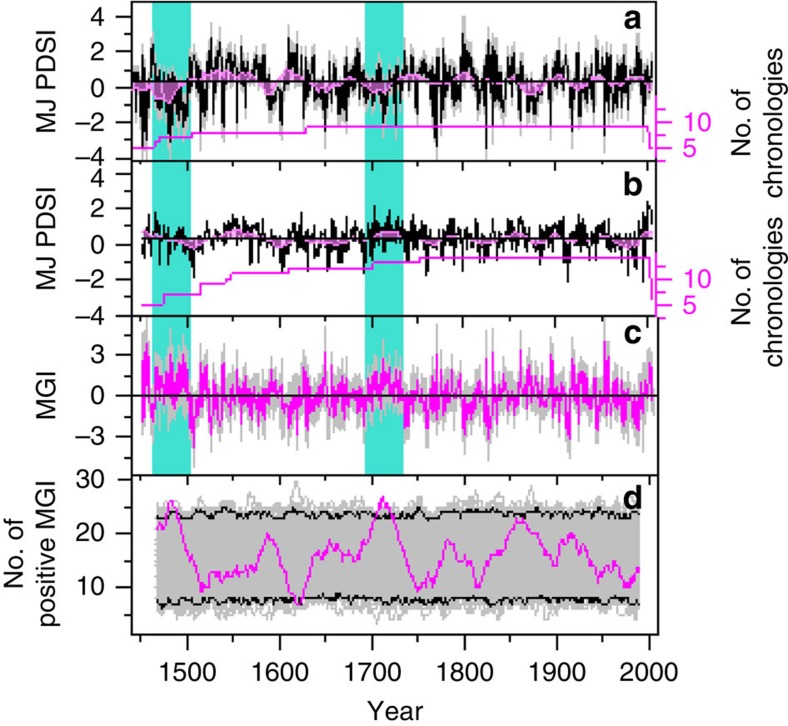
Spatiotemporal patterns in May–June PDSI on the TP for the past five and a half centuries. Reconstruction of May–June PDSI (curve in black) from network of tree-ring chronologies (the number of component site chronologies is shown) spans 1,442–2,005 CE for the northern TP (**a**) and 1,451–2,006 CE for the southern TP (**b**). Moisture gradient index (MGI, southern reconstruction minus northern reconstruction) between the two regions is calculated (curve in pink) (**c**), and the calculation also includes the number of positive MGI in a 31-year window (the value is positioned in the 16th year of the window) sliding over the past five and a half centuries (curve in pink) with a background (in grey) of the same calculation but derived from 1,000 first-order autoregressive simulations of the reconstructions of both regions (**d**). The time series of moisture reconstructions in **a** and **b** are smoothed by a 20-year Fast Fourier Transform filter. The grey shading in curves **a**, **b** and **c** denotes 95% confidence interval for prediction of the mean values. The upper and lower borders in **d** indicate 99% range of the 1,000 simulated values. The green bars indicate two climate states of prolonged south–north moisture dipole.

The regional tree-ring width chronologies are each significantly responsive to regionally averaged PDSI in May and June^17^,^23^,^24^ (Fig. 3), and also to the averaged May–June PDSI (*r*=0.66, *N*_eff_=53, *P*<0.001 for the northern region, and *r*=0.72, *N*_eff_=48, *P*<0.001 for the southern region) for the interval 1953–2005 during which instrumental records are available for reliable estimates of PDSI. The first differences (value at year *t* minus value at year *t*−1) of each chronology and May–June PDSI are also significantly correlated (*r*=0.63, *N*=52, *P*<0.001 for the northern region and *r*=0.61, *N*=52, *P*<0.001 for the southern region). Significant correlation of the regional chronologies with regional averages of Vaganov–Shashkin-Lite (VS-Lite) simulations^21^ also support the interpretation of regional variations in tree-ring widths in terms of regional May–June moisture availability (Supplementary Fig. 4). Therefore, we reconstruct the history of May–June PDSI variation from the tree-ring chronologies for the northern and southern regions using regression analysis (see Methods section). A leave-one-out calibration and validation method was used to develop and test the regression models^25^. The statistics of the sign tests and the reduction of error tests indicate that the transfer function is reliable for reconstructing regional PDSI from tree-ring chronologies (Supplementary Table 2) and that individual chronologies are all significantly correlated with the regional reconstructions (Supplementary Table 3). The reconstructed May–June PDSI (Fig. 4a,b) represents 42.5% (50.8%) of the variance in actual PDSI over the period 1953–2005 for the northern (southern) region.

### A moisture dipole on the Tibetan Plateau

Given the spatial structure of the moisture regimes, we calculated the difference of the reconstructed PDSI between the two regions (values in the south minus the north), and define it as a moisture gradient index (MGI; Fig. 4c). To identify the states and fluctuations in the spatial pattern of moisture variability, we generated 1,000 simulations of the reconstructed PDSI time series using a first-order autoregressive model for each region and calculated MGI time series for each pair of simulations. We then examined the number of positive MGI events, and the mean of MGI values exceeding the 99th percentile uncertainty likelihoods in a 31-year window sliding over the past five and a half centuries (Fig. 4d, Supplementary Fig. 5). Two prolonged states of anomalous south–north moisture dipole stand out in 1,463–1,502 CE and 1,693–1,734 CE, and have no analogue in the instrumental period. In addition, we identify extreme anomalous dry or wet years in the northern and southern regions throughout the past five and a half centuries (Supplementary Table 4).

## Discussion

Our study significantly increases the number of chronologies and spatial coverage across the eastern Tibetan Plateau, particularly for the southern region where old-growth forests are located in remote and hard-to-access areas. In the northern region, our moisture reconstruction is significantly correlated with that from the corresponding gridpoints in the MADA^16^ (*r*=0.84, *P*<0.001 *N*_eff_=507 in the period 1442–2005 CE). In the southern region, agreement with MADA estimates is intermittently significant through the period 1451–2005 CE (Supplementary Fig. 6). This disagreement with the MADA in the southern region could arise partly from the difference in reconstructed season (June–August versus May–June), reconstruction methods and number of component chronologies. Here we interpret the updated regional reconstructions as suggesting that the impact of tropical–extratropical interaction on moisture delivery is different between regions and over time. The spatial structure of the tree-ring network shows that the moisture regime division at ∼33° N, as suggested by previous studies^5^,^6^,^8^,^9^, is generally a feature of the past five and a half centuries. This spatial structure also appears in MADA data for the same period (Supplementary Fig. 7). Wavelet coherency spectra for our northern and southern region reconstructed May–June PDSI time series do not show persistent coherency at any resolved periodicity, suggesting that moisture variability in the northern and southern Tibetan Plateau is not uniform through time (Supplementary Fig. 8). Climate reconstructions from numerous localized studies in the northeastern Tibetan Plateau indicate severe droughts during the second half of the 15th century and the early 18th century^26^,^27^,^28^,^29^. Although these droughts are replicated in our PDSI reconstructions for the northern region (Fig. 4a), they are not apparently dry in our reconstructions for the southern region (Fig. 4b).

The prolonged south–north moisture dipole in 1,463–1,502 CE and 1,693–1,734 CE may reflect a change in Tibetan Plateau climate related to tropical–extratropical interactions. In general, the westerly wind retreats from south to north during May and June, and the monsoon is strengthening as surface heating increases across the southern plateau^30^,^31^. Bifurcation of the westerly wind due to the orographic effect of the plateau promotes development of cyclonic flow in the south, bringing moisture to the southeastern region, whereas the northern branch forms anticyclonic flow, leading to dry conditions in the northeastern region^9^. The prolonged south–north moisture dipole during which the north was anomalously drier than the south might reflect a climate state in which the westerly wind retreats slowly northward on the Tibetan Plateau and the moisture delivery was primarily related to the mid-latitude Westerlies^32^,^33^. The transition to such climate state might be related to cold episodes in the Little Ice Age, which could slower the northward movement of the Westerlies in spring and suppress strength of the monsoon by reducing the land–sea thermal gradient^34^. Frequent occurrence of strong El Nino events during 1,693–1,734 CE, reconstructed from multiple independent paleoclimatic observations, is also consistent with a weak monsoon at these times^35^,^36^.

Years during which the south was anomalously dry and the north was anomalously wet (Supplementary Table 4, second column) may suggest the presence of strong moisture transport by mid-latitude Westerlies to the northern region, and a weak early monsoon with strong evapotranspiration in the southern region^4^. Although moisture delivery anomalies in this relatively dry region may be determined by a few transient events, the resulting moisture anomalies are expected to be integrated into a soil moisture deficit to which tree growth responds over the course of the growing season^21^,^37^. Extreme anomalously dry or wet years common in both the southern and northern regions (Supplementary Table 4, third and fourth columns) suggest that the tropical and extratropical systems acted to produce similar moisture anomalies across the eastern plateau. For example, the severe droughts in both regions in 1,602 CE were possibly associated with the 1,600 CE Huaynaputina eruption^38^ in Peru, which is likely to have resulted in globally decreased water content in the atmosphere^39^ and consequently reduced moisture delivery to the Tibetan Plateau by both the South Asian Monsoon and mid-latitude Westerlies. More generally, however, the MGI shows interannual fluctuations around zero throughout most of the record. These variations in the MGI are very likely to arise from stochastic, unforced interaction between the Westerlies and the monsoon circulation rather than from a steady and single dominant mechanism.

Our tree-ring records of moisture variability on the southern and northern Tibetan Plateau do not provide direct evidence of moisture sources for the south and the north Tibetan Plateau, rather they provide a basis for detecting variations in moisture delivery to the Tibetan Plateau by the South Asian Monsoon and the mid-latitude Westerlies over the past five and a half centuries. Extended moisture dipole events that appear within the Little Ice Age temporally overlap with the Spörer (1,460–1,550 CE) and Maunder (1,645–1,715 CE) minima in solar activity^40^,^41^, thus providing important targets for simulation of the statistics of persistent hydroclimatic changes in response to external radiative forcing^42^,^43^ or as a feature of the unforced variation^44^ (Supplementary Fig. 8). In periods without pronounced external radiative forcing, variations are evident in the amplitude, frequency and duration of dipole activity, likely arising stochastically from interaction of the tropical and extratropical circulation. Our study extends our knowledge of the observed interannual–decadal dipole variations in hydroclimate over the eastern Tibetan Plateau for several centuries, and may facilitate further studies of dynamical interactions between the Asian monsoon circulation and the extratropical Westerlies.

## Methods

### Tree-ring chronology development

Tree-ring widths of the samples were measured to the nearest 0.001 mm using a Lintab system (Frank Rinntech Company, Heidelberg, Germany). Each tree-ring was assigned a calendar year of its formation by means of crossdating, a technique that is based on the recognition that trees growing in the same period and under the same climate condition exhibit similar changes in the year-to-year growth^45^. The crossdated tree-ring series were then standardized and averaged to produce a site standard tree-ring chronology. In the process of tree-ring standardization, growth trends due to non-climatic effects (for example, aging and stand dynamics) were removed using a cubic spline of 50% frequency-response cutoff at half length of each series^46^. Because the samples have different ages, the years in the earlier portion of the resulting chronology are represented by fewer samples. Thus, we truncated the length of each chronology to represent years that have at least five replicate ring-width measurements. The expressed population signal^47^, which represents the strength of the common signal across tree-ring series entering a chronology, averages 0.78 for the 23 site chronologies in their earliest 50 years consisting of five samples. Because the northern and southern regional chronologies in this study are composed of at least five site chronologies, the sample replication is sufficient for the regional chronologies.

### Growth–climate relationships

Correlation analysis was used to identify the relationships between tree-ring chronologies and climatic variables. The climatic variables included mean monthly temperature and total monthly precipitation from weather stations nearest to the tree-ring sampling sites. We also examined the relationships between site tree-ring chronologies and PDSI^17^ at 2.5° × 2.5° grids covering the sampling sites. The relationship between regionally averaged tree-ring chronologies and the regional PDSI was identified using DendroClim2002 programme^48^.

The statistical growth–climate relationships identified in this study were compared with those inferred from process-based forward simulations using the VS-Lite model^21^ of tree-ring width variations. The model simulates tree-ring chronologies by integrating a monthly resolution nondimensional growth response to either air temperature or soil moisture, depending within each time step on which is the limiting factor, the result scaled by insolation (as estimated from the latitude of the study site). Soil moisture is simulated in VS-Lite from input monthly temperature and accumulated precipitation via the CPC Leaky Bucket model^37^. In this study, we simulated ring widths using climate inputs in May–June for the period 1953–2000 for each of the 23 sites and for regions of the southern and northern Tibetan Plateau.

### Reconstruction of past May–June PDSI

Transfer functions for the north and south regions were developed using linear regression techniques, in which the dependent variable is regional May–June PDSI and the independent variable is regional tree-ring chronology. The regional May–June PDSI is obtained by averaging the PDSI series over the six grids for the north region and over the five grids for the south region in the period 1953–2005. The regional tree-ring chronology is obtained by standardizing site chronologies with respect to period 1951–2000 and averaging the standardized site chronologies over the 9 sites for the north region and over the 14 sites for the south region. The transfer functions are as follows.


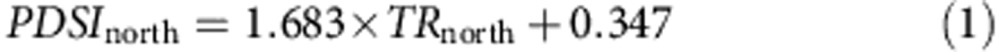



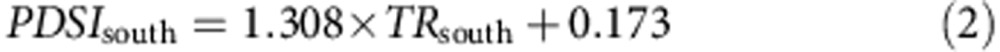


Where PDSI indicates the regional drought index, TR is the regional tree-ring width chronology, and subscripts indicate north or south region of the Tibetan Plateau. Calibration and validation statistics over the period 1953–2005 demonstrate that these two regression models are reliable (Supplementary Table 2).

## Additional information

**How to cite this article**: Zhang, Q. B. *et al*. Moisture dipole over the Tibetan Plateau during the past five and a half centuries. *Nat. Commun.* 6:8062 doi: 10.1038/ncomms9062 (2015).

## Supplementary Material

Supplementary Information Supplementary Figures 1-8 and Supplementary Tables 1-4
