# Supplementary material for: Moisture dipole over the Tibetan Plateau during the past five and a half centuries
Source: Nat Commun. 2015 Aug 21;6:8062. doi: 10.1038/ncomms9062 (PMC4560780; doi:10.1038/ncomms9062)
Supplement: Supplementary Information — Supplementary Figures 1-8 and Supplementary Tables 1-4 [file ncomms9062-s1.pdf]

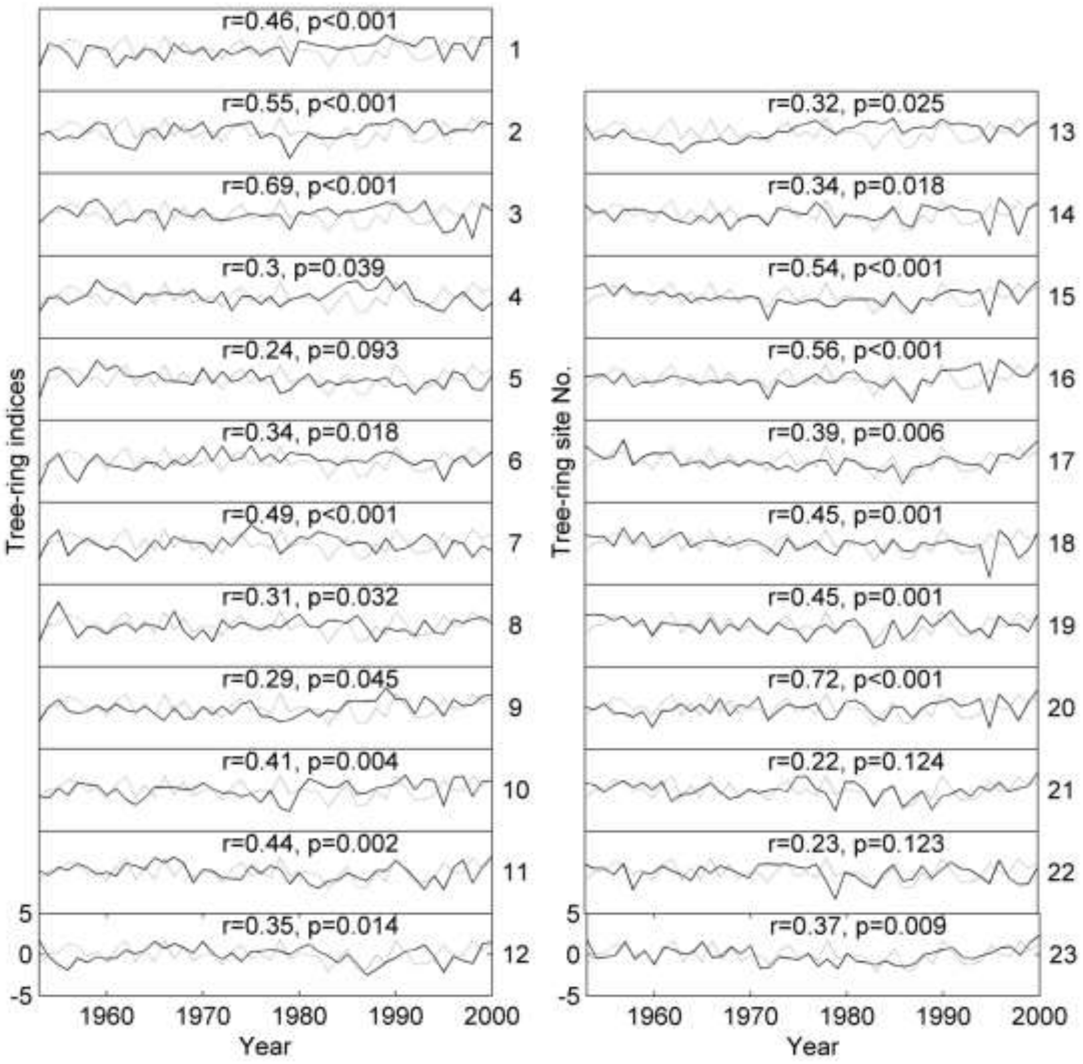

Supplementary Figure 1. Comparisons of actual tree-ring chronologies (solid line) with simulated chronologies from VS-Lite modeling (dashed line) for each site in the period 1953-2000. The inputs to the model include latitude of the study site, monthly mean temperature and monthly total precipitation in May and June from closest weather station. The tree-ring site numbers are arranged in the order of latitude from north to south and are consistent with those in Fig. 1 and supplementary Table 1.

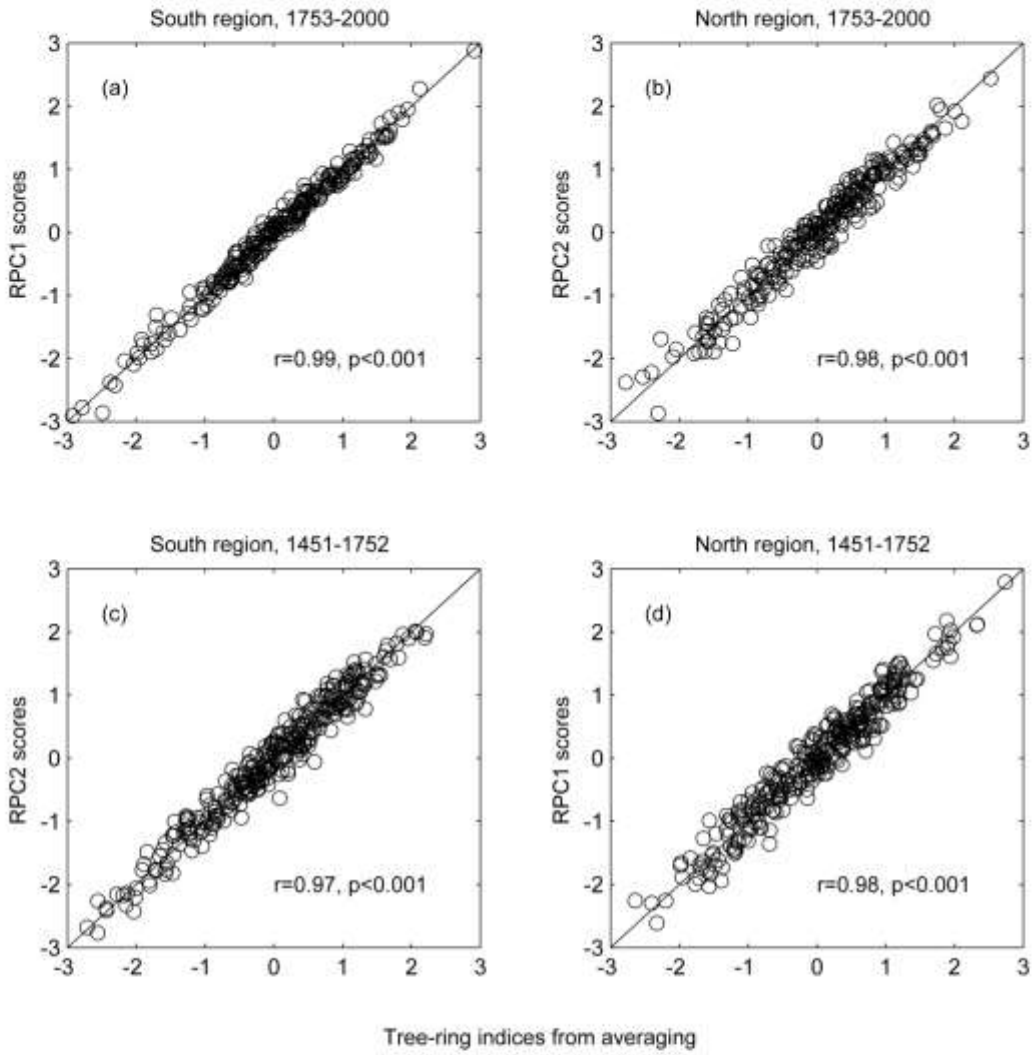

Supplementary Figure 2. Comparisons of the site-averaged regional tree-ring chronologies with the PC scores from rotated EOF. South regional chronology and PC1 scores (a), north regional chronology and PC2 scores (b) for the 23 site chronologies in their common period 1753-2000. South regional chronology and PC2 scores (c), north regional chronology and PC1 scores (d) for the 10 longest site chronologies in the period 1451-1752.

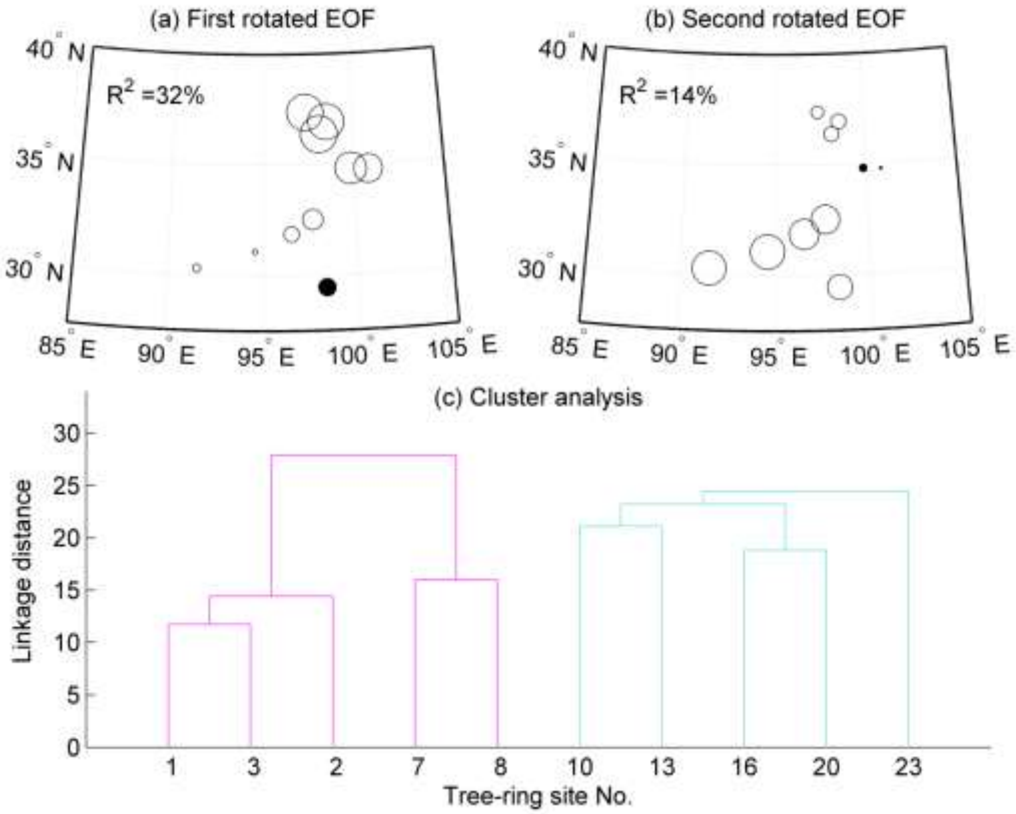

Supplementary Figure 3. Spatial structure of the 10 longest site chronologies in the period 1451-1752 CE. The size of circles represents value of loadings for sites in the first rotated EOF (a) and the second rotated EOF (b) (the filled circle represents value of opposite sign). Cluster analysis shows linkage relations of the northern sites (coloured in red) and southern sites (coloured in green) (c). This structure indicates persistent spatial pattern as shown in the full 23 site chronologies in their common period 1753-2000 CE. The tree-ring site number are arranged in the order of latitude from north to south and are consistent with those in Fig. 1 and supplementary Table 1.

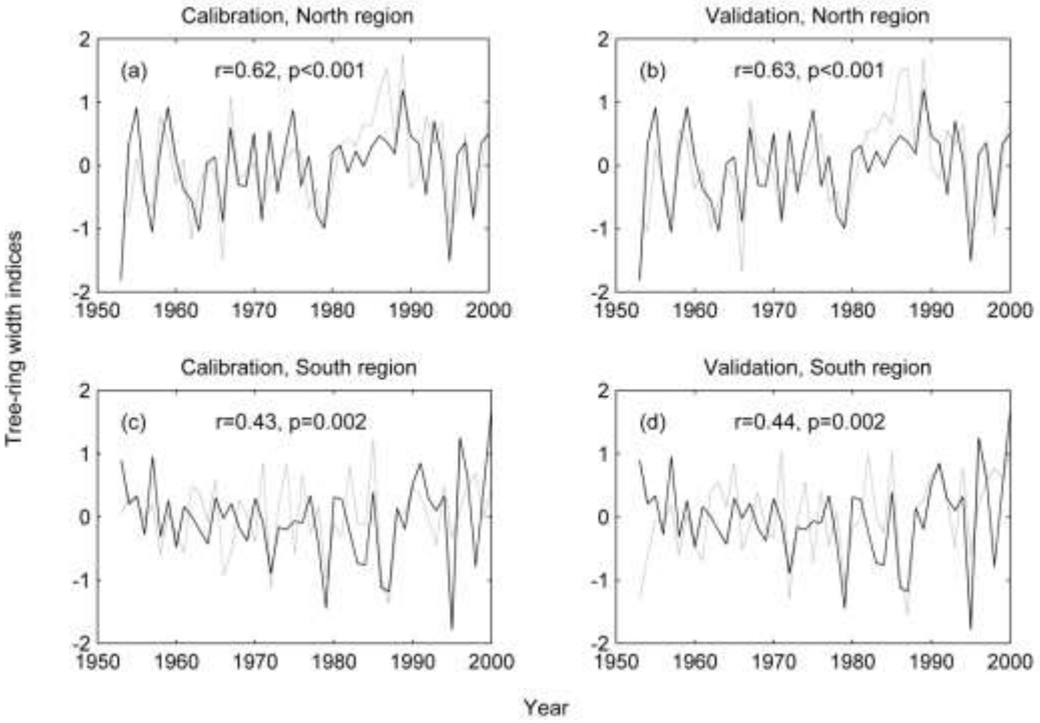

Supplementary Figure 4. Comparisons of the north and south regional tree-ring chronologies (solid line) with simulations of VS-Lite modeling (dashed line) in period 1953-2000. The regional simulations are mean time series of the component site simulations from 100 repeated experiments with a random 24-year subset for calibration and the rest half subset for validation using May-June temperature and precipitation as input climate variables. North region calibration (a) and validation (b), and south region calibration (c) and validation (d).

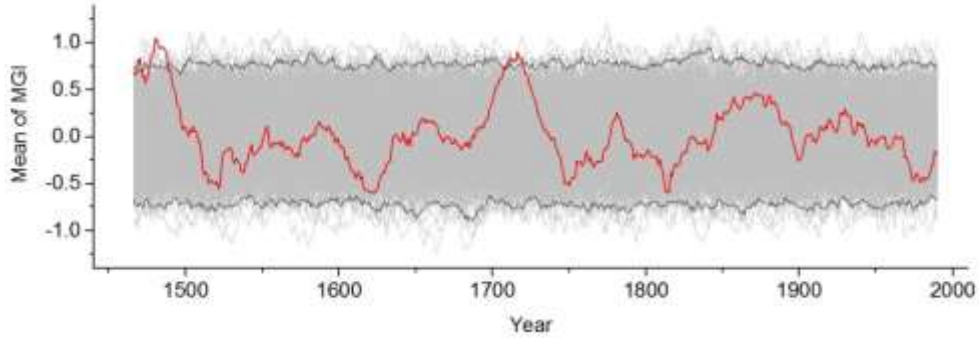

Supplementary Figure 5. Mean of south-north regional moisture gradient index (MGI) in a 31-year window sliding over the past five and a half centuries (curve in red) with a background of the same calculation but derived from 1000 first-order autoregressive simulations of the reconstructions of both regions. Values in the 31-year windows are positioned in the 16th year of the window. The upper and lower borders indicate 99% range of the 1000 simulated values so that the red curve exceeding the envelope indicates intervals that the sum of MGI is significant at $p<0.01$.

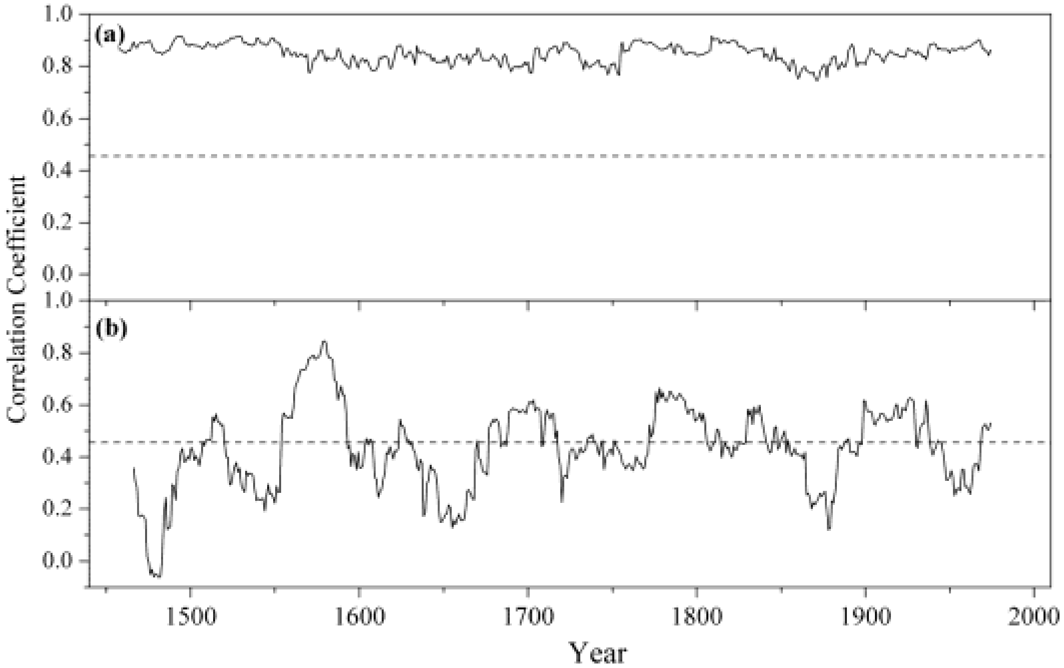

Supplementary Figure 6. Correlation coefficients between the reconstructed PDSI of this study and that of the MADA in a 31-year running window during 1451-2005 CE for the same grids in the northern TBP (a) and southern TBP (b). The correlation coefficients were placed in the middle year of the window. The dashed lines refer significance level at $p<0.01$.

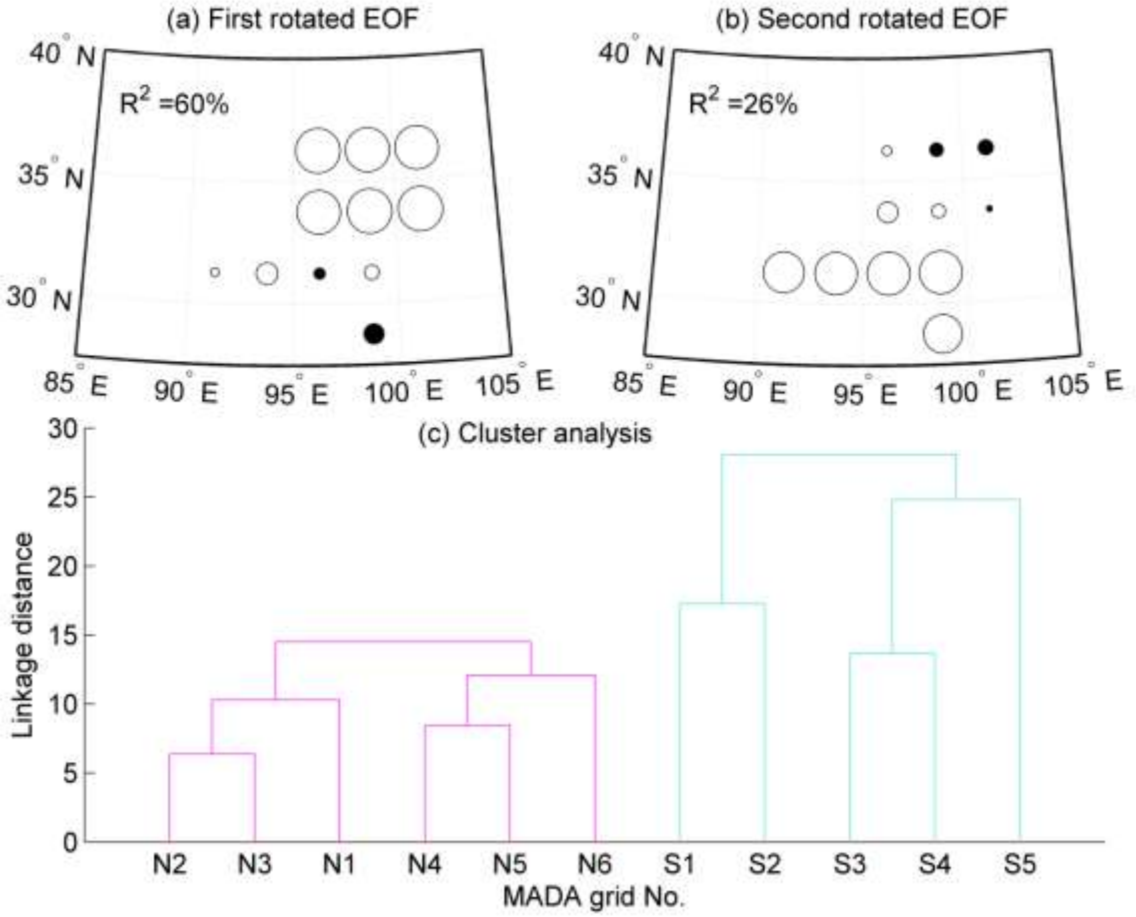

Supplementary Figure 7. Spatial structure of the PDSI reconstructions at the 11 grids same as this study but from MADA data in the period 1451-2005. The size of circles represents relative loadings for grids in the first rotated EOF (a) and the second rotated EOF (b) (the filled circle represents value of opposite sign). Cluster analysis shows linkage relations of the northern grids (coloured in red) and southern grids (coloured in green) (c). The northern six grids (N1 to N6) and the southern five grids (S1 to S5) are labelled from northwest to southeast.

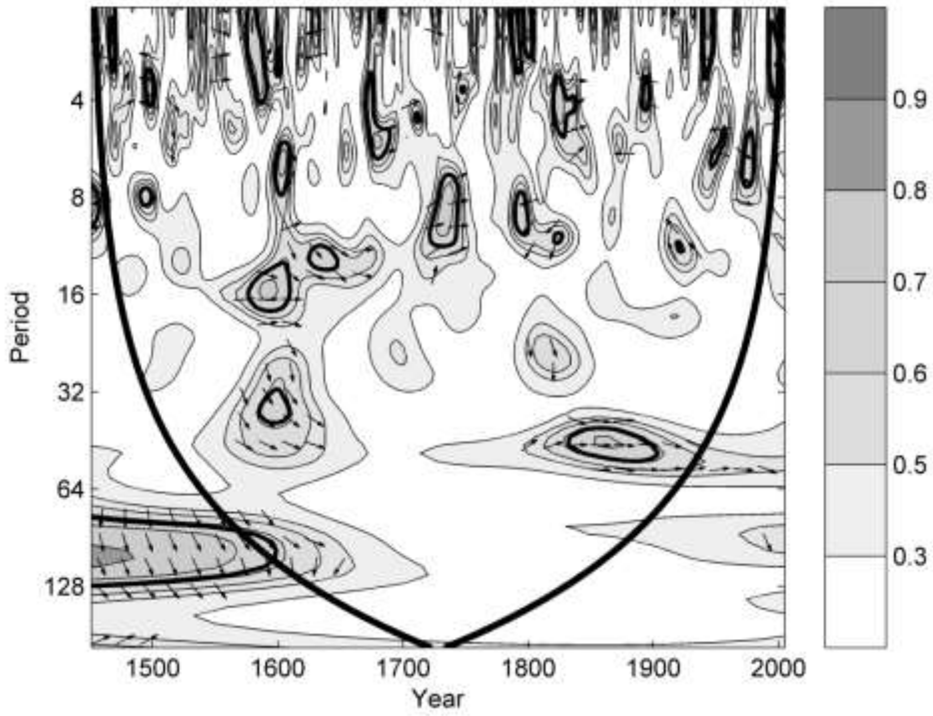

Supplementary Figure 8. Wavelet coherency between the reconstructed May-June PDSI in the southern and northern TP. The legend indicates squared coherency spectrum and the thick black contour is the significance level at $p < 0.05$ against red noise spectra. The arrows indicate phase relationships between the two series (in-phase pointing right, anti-phase pointing left). The regions outside the curves on either end indicate the "cone of influence", where edge effects become important relative to period of variation.

Supplementary Table 1. Information about the 23 tree-ring chronologies on the Tibetan plateau.

Site No.	Site name	Latitude (N)	Longitude (E)	Elevation (m)	Chronology length	Number of trees	Mean R_{bt}	$R_{chron\text{-}PDSI}$
1	Delingha*	37.37	97.37	3820	976 - 2000	37	0.76	0.56^{a}(47)
2	Wulan*	36.94	98.55	3640	1322 - 2001	22	0.58	0.32^{d}(30)
3	Dulan*	36.37	98.13	3610	278B.C.E.– 2000C.E.	88	0.68	0.47^{b}(40)
4	Tongren	35.83	102.02	3260	1630 - 2001	31	0.43	0.42^{b}(40)
5	Zhongtie	35.05	100.1	3831	1470-2008	31	0.55	0.27^{d}(45)
6	Jiangqun*	35.02	100.35	3626	1465 - 2005	28	0.46	0.29^{d}(45)
7	Xueshan	34.8	99.84	3644	1320 - 2005	26	0.65	0.50^{a}(48)
8	Hebei*	34.76	100.81	3320	1442 - 2005	27	0.50	0.32^{c}(48)
9	Ningmute	34.62	101	3575	1506-2008	34	0.52	0.25^{d}(48)
10	Dongzhong	32.52	97.67	4018	1396 - 2001	31	0.51	0.49^{a}(45)
11	Dongba	32.19	95.64	4192	1515 - 2001	29	0.67	0.31^{c}(45)
12	Jiangxi	32.07	97.07	3637	1515 - 2001	39	0.56	0.27^{d}(43)
13	Baizha	31.87	96.52	3908	1378 - 2001	32	0.55	0.28^{d}(47)
14	Suoxian*	31.63	94.29	3854	1753 - 2004	27	0.45	0.39^{b}(48)
15	Biru	31.12	93.87	4350	1475 - 2005	31	0.55	0.49^{a}(48)
16	Bianbamx	31.08	94.58	4144	1449 - 2006	24	0.62	0.53^{a}(48)
17	Gongjue	30.75	98.69	3817	1475 - 2006	24	0.46	0.51^{a}(45)
18	Jiali*	30.6	93.46	4250	1542 - 2004	30	0.61	0.47^{a}(48)
19	Luolong	30.58	96.18	4440	1548 - 2006	32	0.74	0.42^{b}(47)
20	Linzhou	30.31	91.51	4233	1442 - 2004	30	0.58	0.64^{a}(48)
21	Basu	30.06	97.12	4382	1702 - 2006	29	0.54	0.32^{c}(46)
22	Gbjda	29.82	92.65	4250	1611 - 2004	23	0.53	0.39^{b}(48)
23	Mangkang	29.45	98.35	4050	1451 - 2006	28	0.55	0.49^{a}(48)

Site No. is listed in the order from north to south; star sign (*) indicates sites previously reported on journals; Mean R_{bt} refers to mean inter-serial correlation; $R_{chron\text{-}PDSI}$ refers to correlation coefficient between site tree-ring chronology and May-June PDSI in the interval 1953-2000, in which ^{a}, ^{b}, ^{c} and ^{d} indicate the significance lever at $p<0.001$, $p<0.01$, $p<0.05$, and $p<0.1$, and the numbers in the parentheses are effective sample size.

Supplementary Table 2. Calibration and validation statistics for the two regression models over the period 1953-2005.

Calibration				Validation				
Model	*r*	R^2	R^2_{adj}	ST	ST1	*r*	PMT	RE
North	0.66	43.6%	42.5%	36+/17–*	37+/15–**	0.63	3.58	0.40
South	0.72	51.8%	50.8%	38+/15–**	35+/17–*	0.70	4.38	0.48

r represents correlation coefficient; R^2 indicates variance in May-June PDSI explained by tree-ring indices; R^2_{adj} indicates explained variance after adjustment for degree of freedom; ST (sign test) indicates the degree that tree rings track the direction of year to year change in climate; ST1 refers to first difference sign test; PMT (product mean test) is a measure of the sign and magnitude of departure from the calibration mean; RE (reduction of error) is a measure of explained variance between actual and estimated series, with a positive value suggesting an encouraging model performance; ** and * indicate the significance level at $p<0.01$ and $p<0.05$, respectively.

Supplementary Table 3. Correlation between the site tree-ring chronologies and their corresponding regional May-June PDSI reconstructions on the eastern Tibetan plateau.

Site No.	Site name	Correlation coefficient	P values	Number of years	Interval of comparison
1	Delingha	0.71	<0.0001	559	1442 - 2000
2	Wulan	0.69	<0.0001	560	1442 - 2001
3	Dulan	0.68	<0.0001	559	1442 - 2000
4	Tongren	0.36	<0.0001	372	1630 - 2001
5	Zhongtie	0.57	<0.0001	536	1470 - 2005
6	Jiangqun	0.64	<0.0001	541	1465 - 2005
7	Xueshan	0.70	<0.0001	564	1442 - 2005
8	Hebei	0.64	<0.0001	564	1442 - 2005
9	Ningmute	0.60	<0.0001	500	1506 - 2005
10	Dongzhon[illegible]	0.52	<0.0001	551	1451 - 2001
11	Dongba	0.57	<0.0001	487	1515 - 2001
12	Jiangxi	0.52	<0.0001	487	1515 - 2001
13	Baizha	0.50	<0.0001	551	1451 - 2001
14	Suoxian	0.53	<0.0001	252	1753 - 2004
15	Biru	0.66	<0.0001	531	1475 - 2005
16	Bianbamx	0.62	<0.0001	556	1451 - 2006
17	Gongjue	0.43	<0.0001	532	1475 - 2006
18	Jiali	0.64	<0.0001	463	1542 - 2004
19	Luolong	0.54	<0.0001	459	1548 - 2006
20	Linzhou	0.60	<0.0001	554	1451 - 2004
21	Basu	0.51	<0.0001	305	1702 - 2006
22	Gbjda	0.59	<0.0001	394	1611 - 2004
23	Mangkang	0.45	<0.0001	556	1451 - 2006

Supplementary Table 4. Top 10 extreme years that the May-June PDSI was in greatest contrasting or similar conditions in the northern and southern Tibetan Plateau during 1451-2005 CE.

Moisture in contrast		Moisture in common	
North dry south wet	North wet south dry	Both dry	Both wet
1455	1446	1451	1544
1458	1504	1480	1549
1495	1507	1602	1618
1649	1631	1665	1624
1713	1760	1672	1673
1831	1827	1789	1683
1861	1914	1824	1744
1895	1944	1979	1832
1953	1986	1995	1863
1957	1987	1998	1951
